# Recent Advances in Porous Carbon Materials as Electrodes for Supercapacitors

**DOI:** 10.3390/nano13111744

**Published:** 2023-05-26

**Authors:** Zhengdao Pan, Sheng Yu, Linfang Wang, Chenyu Li, Fei Meng, Nan Wang, Shouxin Zhou, Ye Xiong, Zhoulu Wang, Yutong Wu, Xiang Liu, Baizeng Fang, Yi Zhang

**Affiliations:** 1 School of Energy Sciences and Engineering, Nanjing Tech University, Nanjing 211816, China; 2 Department of Chemistry, Washington State University, Pullman, Washington, DC 99164, USA; 3 Kucap Smart Technology (Nanjing) Co., Ltd., Nanjing 211106, China; 4 Department of Energy Storage Science and Technology, University of Science and Technology Beijing, 30 College Road, Beijing 100083, China

**Keywords:** porous carbon, electrodes, supercapacitors, electrochemical performance

## Abstract

Porous carbon materials have demonstrated exceptional performance in various energy and environment-related applications. Recently, research on supercapacitors has been steadily increasing, and porous carbon materials have emerged as the most significant electrode material for supercapacitors. Nonetheless, the high cost and potential for environmental pollution associated with the preparation process of porous carbon materials remain significant issues. This paper presents an overview of common methods for preparing porous carbon materials, including the carbon-activation method, hard-templating method, soft-templating method, sacrificial-templating method, and self-templating method. Additionally, we also review several emerging methods for the preparation of porous carbon materials, such as copolymer pyrolysis, carbohydrate self-activation, and laser scribing. We then categorise porous carbons based on their pore sizes and the presence or absence of heteroatom doping. Finally, we provide an overview of recent applications of porous carbon materials as electrodes for supercapacitors.

## 1. Introduction

**Figure 1:**
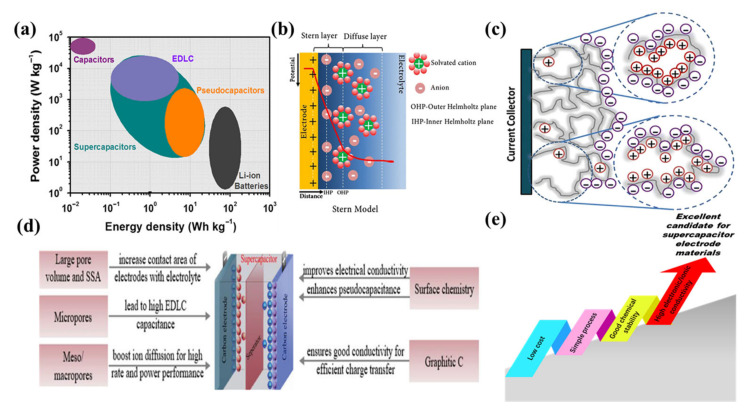
(**a**) Ragone plot of different energy storage devices. Reproduced with permission from Ref. [6]. Copyright 2019 Zhengzhou University. (**b**) Representation of an EDLC structure: Gouy–Chapman–Stern model. (**c**) Schematic illustration of charge storage mechanism of EDLC in porous carbon electrode. Reproduced with permission from Ref. [40]. Copyright 2020 Springer. (**d**) Structure-dependent properties of porous carbons as electrode materials in supercapacitors. Reproduced with permission from Ref. [44]. Copyright 2020 WILEY-VCH Verlag GmbH. (**e**) Advantages of porous carbons as an electrode for supercapacitors.

With the development of science and technology and the progress of human civilisation, our use of energy has increased significantly, which has led to the gradual depletion of fossil fuels and a year-on-year rise in carbon dioxide emissions [1,2,3,4,5]. The development of new renewable energy sources, such as solar, wind, and nuclear power, has become a hot topic of the times [6,7,8,9,10,11]. Based on this, it is vital to research and create new high-performance green energy storage and conversion devices [12,13,14,15,16]. At present, research into and applications of energy storage devices are mainly focused on various types of batteries and supercapacitors [17,18,19,20,21,22,23,24,25,26,27,28,29,30,31]. Among other things, supercapacitors have excellent properties such as high power density, the ability to charge quickly, long service life, good environmental protection, and high energy efficiency [32,33]. However, their biggest drawback compared to today’s commercially mature lithium-ion batteries is their lower energy density (Figure 1a) [6,34,35,36,37,38,39]. This problem is even more pronounced in the case of double-layer supercapacitors. The reason for this is that double-layer supercapacitors store energy only through the adsorption of electrolyte ions onto the surface of the active material (Figure 1b) [40,41]. This non-Faraday, dual-layer energy storage mechanism and lack of volume expansion make it difficult to obtain a high energy density for dual-layer supercapacitors, although they can provide higher power density and lifetime than batteries, which is the bottleneck for their large-scale use, and porous carbon electrodes are the key to break the bottleneck.

The pores of porous carbon materials enable rapid adsorption and desorption of electrolyte ions at the electrode/electrolyte interface (Figure 1c) [40,42]. In addition, the capacitance of a double-layer supercapacitor is directly related to the contact area between the electrodes and the electrolyte, so a sufficient amount of porosity is a fundamental requirement for a high-performance double layer supercapacitor [43]. In principle, increasing specific surface area (SSA), pore volume, and conductivity of porous carbon electrodes can effectively enhance charge accumulation in electric double-layer formations, resulting in greater double-layer supercapacitor capacitance. However, some studies have found that ultra-high SSA may also lead to a decrease in bulk capacitance due to the relatively low stacking density. It is therefore important to control the pore size appropriately to maximise the achievable SSA and reduce the dead volume (Figure 1d) [44,45,46]. Most importantly, the low cost of porous carbon materials can reduce the cost of energy storage devices, and the porous carbon preparation process is simple, with good chemical stability and high electronic conductivity [47,48,49]. These advantages determine porous carbons as a suitable electrode material for supercapacitors (Figure 1e).

This paper reviews the synthesis of porous carbon materials and their application in supercapacitor electrodes. The overall content is as follows: Firstly, we present the different methods for the synthesis of porous carbons in three categories. Then, the application of porous carbons in supercapacitor electrodes is divided into two sections: heteroatom-free doped porous carbons and heteroatom-doped porous carbons, each of which is classified according to the pore size of the porous carbons and their applications are described in detail. Finally, we conclude with a summary and outlook in this review. Unlike previous reviews by other scholars who introduced porous carbons as electrodes for supercapacitors, this review not only introduces the most common and novel methods for preparing porous carbons, but also clearly explains the application of different types of porous carbons as supercapacitor electrodes in recent years through a more detailed classification compared with previous reviews.

## 2. Synthesis of Porous Carbon Materials

The synthesis of porous carbon materials is mainly divided into carbonisation-activation methods and template methods. In addition, there are some other methods.

### 2.1. Carbonisation–Activation Methods

A combined carbonisation-activation method is frequently used to prepare porous carbons. The summary of various methods is shown in Table 1, and will be introduced in detail in this section.

In general, carbonisation is achieved by charring the organic precursors at 400–1000 °C in an inert atmosphere. Through carbonisation we can obtain non-porous solid carbon called coal or biochar [50,51]. A pore-forming agent is used in the activation process to form the pores. The coal or biochar is then activated with an activator (CO_2_, O_2_, air or H_2_O for physical activation, KOH, Na_2_CO_3_, ZnCl_2_, H_3_PO_4_, etc. for chemical activation [52,53]). Physical activation is currently mostly used in industrial production, and the activation of coconut shells using steam as an activator can yield a porous carbon with a SSA of about 1500 m^2^ g^−1^. Chemical activation based on KOH, NaOH and other activators is now more oriented towards laboratory applications, and although some experiments have been able to achieve a high SSA of 500–3600 m^2^ g^−1^, the high cost of activators is still a problem to be solved [54,55,56]. In this subsection, the different synthesis strategies of porous carbons are briefly described in terms of physical and chemical activation methods.

#### 2.1.1. Physical Activation

**Figure 2:**
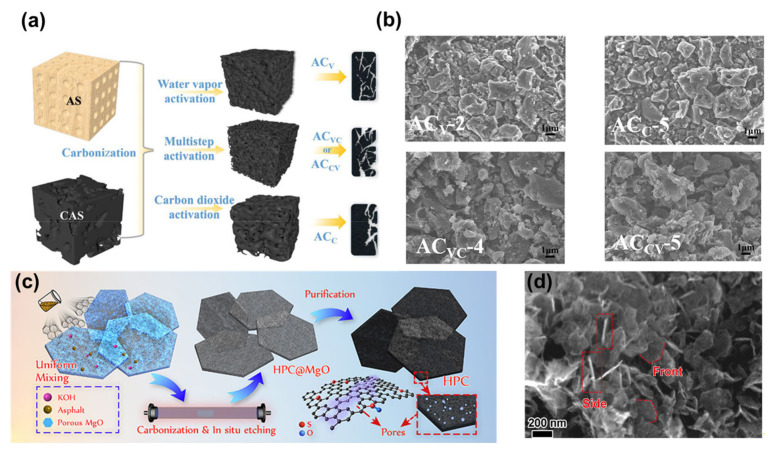
(**a**) Schematic diagram of the preparation of apricot shell-activated carbon. (**b**) SEM images of the activated carbon with different optimisation strategies. Reproduced with permission from Ref. [62]. Copyright 2022 American Chemical Society. (**c**) Schematic diagram of the preparation of HPC. (**d**) SEM image of HPC. Reproduced with permission from Ref. [68]. Copyright 2021 American Chemical Society.

After the material has been carbonised at a high temperature, different activation methods can be used to obtain the pores, and one of the major advantages of treatment by physical activation is that the activator itself is less corrosive to the production equipment. In addition, physical activation has the advantages of being cost effective and environmentally friendly [57,58]. At present, physical activation methods are more suitable for industrial production than chemical activation methods. It has been shown that the physical activation temperature has an approximately linear effect on micropore volume, allowing for more precise adjustment of pore size distribution and thus producing more micropores than chemical activation [53,59,60]. However, it is difficult to obtain porous carbons with a high SSA and rich porosity by single physical activation such as water vapour activation and CO_2_ activation [61]. Ding et al. proposed a multi-step physical activation method using carbonised apricot shells, whereby water vapour pre-activated the carbonised apricot shells to assist in increasing the diffusion rate of CO_2_, resulting in a porous carbon with a high SSA, abundant porosity and a wide pore size distribution (Figure 2a,b) [62]. The best effective pore volumes, mesopore volume fractions and SSAs obtained for the porous carbon reached 0.720 cm^3^ g^−1^, 55.76% and 2400 m^2^ g^−1^, respectively.

#### 2.1.2. Chemical Activation

The high yield of porous carbon and the relatively low temperature of the process compared to physical activation are the reasons why chemical activation is constantly being researched and developed [60,63]. In the latest research, it has been suggested that of the several chemical activators, KOH is the strongest, followed by NaOH [64]. However, according to the chemical activation process, the mass ratio of KOH to carbon precursors for the preparation of porous carbon is generally greater than 3:1 [65,66,67]. This makes the manufacture of porous carbon more expensive and the amount of activator should be further reduced as the activation reaction generates a large amount of K which can easily lead to explosions [68]. A chemical activation method incorporating a template was used by Cao et al. (Figure 2c,d) with a mass ratio of activator to carbon precursor of only 0.5:1, much lower than the conventional dosage [68]. Specifically, the bitumen was converted into hexagonal porous carbon (HPC) with a micro- and mesoporous structure. The SSA of the HPC can reach 1356 m^2^ g^−1^, which is even higher than the porous carbons obtained from many efforts using large amounts of activator, which not only reduces costs but also greatly reduces energy consumption, providing a promising route towards the mass production of porous carbon electrode materials.

**Table 1:** Comparison of porous carbon prepared by different carbonisation-activation methods.

Methods	Basic Steps (Raw Hole Mechanism)	Advantages	Disadvantages	Ref.
Carbonisation—physical activation method	high temperature	Process maturity	Low SSA	[57,58,62]
Carbonisation—chemical activation method	Pore creation by chemical activators	Low temperature	Chemical activators are expensive	[60,63,64,66,67]
Carbonisation—combined physicochemical activation	Combination of high temperature and chemical activators	Safer and more environmentally friendly	High temperature	[65]
Emerging carbonation—chemical activation method	Combining template method with chemical activator to create pores	Less chemical activator usage	Template preparation	[68]

### 2.2. Template Methods

The template method is the most commonly used method for preparing porous carbons in industrial production today. Generally, it can be divided into hard, soft, sacrificial and self-templating methods. The summary of various methods is shown in Table 2, and will be introduced in detail in this section.

#### 2.2.1. Hard-Templating Method

**Figure 3:**
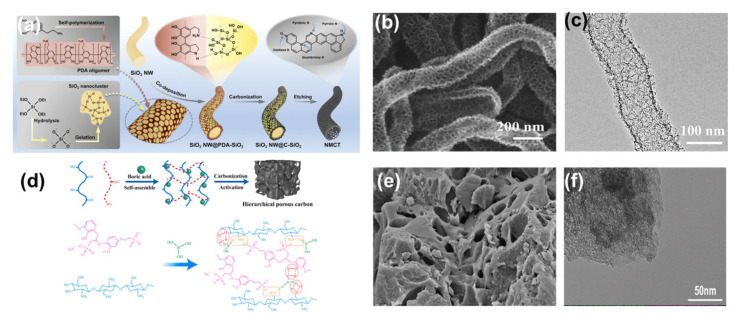
(**a**) Schematic diagram of the synthesis process of the N-doped mesoporous carbon nanotubes. (**b**) SEM image of the N-doped mesoporous carbon nanotubes. (**c**) TEM image of the N-doped mesoporous carbon nanotubes. Reproduced with permission from Ref. [69]. Copyright 2022 Wiley-VCH GmbH. (**d**) Schematic diagram of the synthesis mechanism of layered porous carbon. (**e**) SEM image of the layered porous carbon. (**f**) TEM image of the layered porous carbon. Reproduced with permission from Ref. [80]. Copyright 2022 Elsevier Ltd.

The hard-template method usually uses hard materials such as SiO_2_, MgO and ZnO as template materials, which can be used for synthesis of homogeneous and regular porous structures [69,70,71,72,73,74,75]. The key steps in the preparation of porous carbons by the hard-template method include: (1) preparation of the hard template; (2) impregnation of the hard template with a carbon source; (3) high temperature pyrolysis; and (4) etching of the template with an acid or alkaline solution [53,76]. Of these oxide templates, SiO_2_ is one of the most established templates used for porous carbon synthesis. Zhang et al. used a hard-template method with SiO_2_ as the template and polydopamine as the carbon source to prepare nitrogen-doped porous carbon nanotubes with excellent properties (Figure 3a–c) [69]. We know that carbon nanotubes have a wide range of applications in areas such as catalysis, sensing and energy storage. However, the long tunnel-like pores and the relatively low SSA of carbon nanotubes often limit their performance in certain applications. The authors prepared nitrogen-doped porous carbon nanotubes by co-depositing polydopamine and SiO_2_ nanoparticles on silica nanowire templates. The porous carbon obtained has a high SSA (1037 m^2^ g^−1^) and a large pore volume (3.6 cm^3^ g^−1^). The increases in SSA and pore volume not only provide abundant active sites, but also effectively facilitate mass transfer. SiO_2_ nanomaterials with different particle sizes are now commercially available as a hard-template material for the production of porous carbons. However, inorganic templates are not cheap and must be removed by acid or alkali etching, which hinders their practical application. The hard-template method still requires further research to overcome known difficulties before it can be used more widely in reality.

#### 2.2.2. Soft-Templating Method

The soft-template method usually uses organic molecules as template materials. In a given solvent, the functional groups of the soft template can provide strong interactive forces, such as hydrogen bonding or electrostatic interactions [77,78,79]. Through this interaction the soft template can be easily combined with the carbon source to form a composite and then carbonised to produce porous carbon. The soft-template method is in principle more suitable for the synthesis of porous carbons than the hard-template method, because the soft template does not need to be removed after carbonisation [53]. However, the bigger problem it faces is that the SSA of the porous carbons obtained by the soft-template method alone will not be very high. Therefore, some researchers have attempted to combine the soft-template method with chemical activation. Wang et al. reported a multi-heteroatom-doped three-dimensional (3D) interconnected carbon material, in which the chitosan amino group protonated by boric acid could be self-assembled with the sulfonic acid group of sodium lignosulfonate by electrostatic adsorption and hydrogen bonding (Figure 3d–f) [80]. The addition of KOH solvent to this process achieved a combination of soft templating and chemical activation, further facilitating the formation of a hierarchically porous carbon structure. The as-obtained porous carbon exhibited a high SSA (2700.65 m^2^ g^−1^), which provided a large number of electrochemically active sites for electrolyte adsorption. This work opened up further possibilities for the preparation of porous carbons by the soft-template method.

#### 2.2.3. Sacrificial-Templating Method

**Figure 4:**
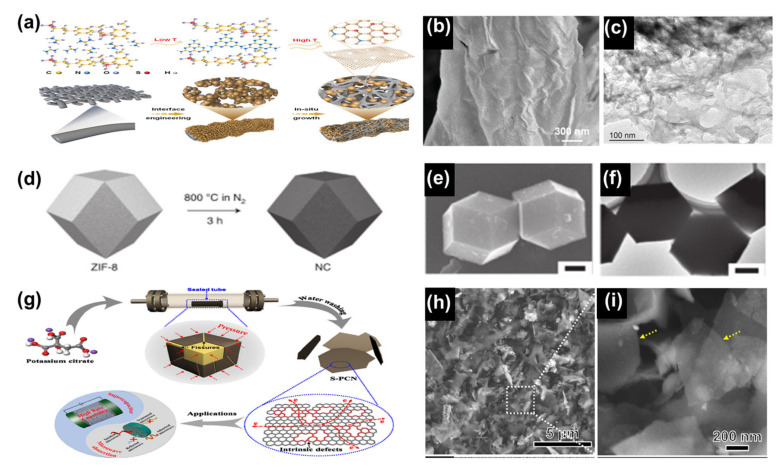
(**a**) Schematic material synthesis and electrode structure engineering of N/S co-doped graphene-like carbon. (**b**) SEM image of N/S co-doped graphene-like carbon. (**c**) TEM image of N/S co-doped graphene-like carbon. Reproduced with permission from Ref. [81]. Copyright 2022 Wiley-VCH GmbH. (**d**) Schematic diagram of ZIF-8 direct carbonisation. (**e**) SEM image of ZIF-8 direct carbonised material. (**f**) TEM image of ZIF-8 direct carbonised material. Reproduced with permission from Ref. [88]. Copyright 2022 Nature. (**g**) Schematic representation of the synthesis of porous carbon nanosheets rich in intrinsic defects and their dual applications. (**h**,**i**) SEM image at 5 μm (**h**) and 200 nm (**i**) magnification of the intrinsic defect-rich porous carbon nanosheets. Reproduced with permission from Ref. [97]. Copyright 2021 Elsevier Ltd.

The advantage of the sacrificial-template method, an emerging template method, is that its templates automatically disintegrate at high temperatures, which is not possible with the hard- and soft-template methods. One substance that fulfils the conditions for a sacrificial template is melamine. Melamine is a template that can be incorporated into a carbon framework. On heating up, it first decomposes into g-C_3_N_4_ and eventually is able to decompose completely [53]. Whereas a major source of melamine is urea, Wang et al. used low-cost urea to form lignosulfonate-derived N/S co-doped graphene carbon in situ within interfacially-engineered cellulose fabrics by sacrificial templating (Figure 4a–c) [81]. Both experimental and theoretical calculations showed that the graphene carbon formed in the pomegranate-like structure had continuous conducting channels and was porous, allowing for adequate ion/electron transport throughout the structure. The lignosulfonates used in this experiment account for the majority of lignin derivatives, with an annual global production of 1.8 million tonnes. The aromatic nature of lignin sulfonates and the sulphonate-containing groups make them the most promising precursors for renewable carbon nanomaterials and they are popular in the sacrificial-template method.

#### 2.2.4. Self-Templating Method

Unlike the template methods described above, the self-templating method is characterised by the fact that it does not require the use of additional porosity agents, but rather the use of self-generated porosity agents from the material [82]. Self-templating materials mainly include MOFs and their derivatives, organic salts, etc. [57,83,84,85,86,87].

The self-templating method is the preparation of porous carbon by direct carbonisation of the self-templating material. The MOFs materials are quite popular in self-templates today. Typical characteristics of MOFs materials include a large SAA, ultra-high porosity, excellent thermal and chemical stability, and great potential for chemical and structural modification, which make them excellent candidates for multifunctional applications. However, their poor electrical conductivity means that their application in electrochemistry is difficult to achieve. Fortunately, some of the carbon atoms in MOFs materials rearrange themselves into a network of carbon atoms after direct carbonisation, thereby enhancing the conductivity of the material [88]. Yamauchi et al. used MOFs particles as a starting material and established a methodology for the synthesis of highly productive and homogeneous porous carbon particles by direct carbonisation of these particles (Figure 4d–f) [88]. The pore size distribution of the obtained porous carbon is highly homogeneous and the SSA can reach 1110 m^2^ g^−1^. The field of research related to the direct carbonisation of MOFs to produce porous carbons has been very active and perhaps MOFs materials could be the key to the future production of low-cost porous carbons [89,90,91].

In addition to MOFs materials, organic salts have also been used in the application of self-templating methods for the preparation of porous carbons. Recent examples of porous carbons obtained by auto-template decomposition of potassium citrate, sodium citrate and calcium citrate at elevated temperatures have produced porous carbons with SSAs of up to 1736 m^2^ g^−1^ [92,93,94,95,96]. Researchers such as Li et al. proposed the idea of introducing inherent defects into the porous carbon skeleton in order to obtain porous carbon with better properties [97]. They prepared porous carbon nanosheets rich in intrinsic defects by using potassium citrate as the only raw material in a sealed tube (Figure 4g–i). The internal pressure inside the sealed tube led to the formation of intrinsic defects, the density of which can be regulated by the volume of the sealed tube. The experimentally-obtained porous carbon nanosheets had a pore structure with a homogeneous distribution of intrinsic defects. This resulted in porous carbon with enhanced wettability and a high SSA.

**Table 2:** Comparison of porous carbons prepared by various template methods.

Methods	Basic Steps (Raw Hole Mechanism)	Advantages	Disadvantages	Ref.
Hard-templating method	Prefabricated template + precursor filling conversion + template removal	Process maturity	Inorganic template is expensive	[69]
Soft-templating method	Co-assembly + surfactant + template removal	No need to add reagents to remove templates	Less control over pore size	[80]
Sacrificial-template method	Co-assembly + sacrificial removal of templates at high temperatures	No need to add reagents to remove templates	Template removal at high temperatures	[81]
Self-templating method	Cracking of ZIFs, MOFs, organic salts, etc.	No template required	Expensive organic salts	[88,97]

### 2.3. Other Methods

In addition to the traditional carbonisation-activation and templating methods, a large number of researchers have worked on several emerging methods for the preparation of porous carbons, some of which are also of great practical value. The summary of these emerging methods is shown in Table 3, and will be introduced in detail in this section.

**Figure 5:**
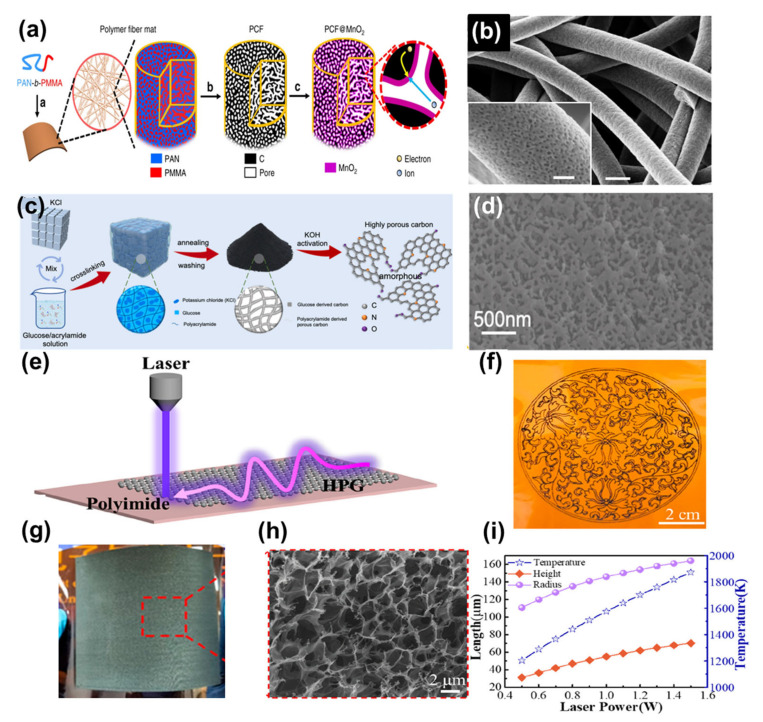
(**a**) Schematic of the synthesis of porous carbon fibres. (**b**) SEM image of porous carbon fibres. Reproduced with permission from Ref. [98]. Copyright 2019 Nature. (**c**) Schematic of the synthesis of porous carbon. (**d**) SEM image of porous carbon. Reproduced with permission from Ref. [102]. Copyright 2023 Elsevier Ltd. (**e**) Schematic of the preparation of honeycomb porous graphene. (**f**) Customised embroidery pattern with honeycomb porous graphene. (**g**) Photograph of flexible honeycomb porous graphene with an area of 8 cm × 8 cm; (**h**) SEM image of honeycomb porous graphene. (**i**) COMSOL simulation results of maximum temperature, carbonisation depth and radius of polyimide at different laser powers. Reproduced with permission from Ref. [106]. Copyright 2021 American Chemical Society.

Conventional carbon-activation methods can prepare porous carbons with controlled SSA and pore size distribution, but require complete removal of the template or activator residue after carbon-activation. As a result, the non-activated approach is becoming increasingly attractive. In a typical organic pyrolysis process, the organic source decomposes and releases gases (e.g., H_2_O, CO_2_, NH_3_ and CO), where the pyrolysis gas acts as a porosity agent [53]. Using the self-assembly of block copolymers (Figure 5a,b), Liu et al. designed porous electrode substrate materials at the molecular level and obtained both of the normally mutually exclusive electrode properties—high loading capacity and fast ion and electron transport capability [98]. The authors synthesised PAN-b-PMMA block copolymers using reversible addition-break chain transfer polymerisation and then converted PAN-b-PMMA into polymer fibres using electrostatic spinning techniques. During the first heating step (280 °C), PAN-b-PMMA underwent phase separation and self-assembly at the nanoscale, resulting in the formation of irregularly bicontinuous PAN and PMMA phases. At the same time, the oxygen in the air promoted ring cross-linking between PAN molecules thus ensuring high carbon yields. Subsequent high-temperature firing carbonised the PAN to form a connected carbon fibre backbone, while the PMMA broke down completely to form interconnected pores. Unlike conventional PAN and carbon fibres prepared by soft and hard template methods, the porous carbon fibres produced by PAN-b-PMMA had uniform pore sizes and interconnected pores evenly distributed throughout the carbon fibre. At a PAN volume fraction of 0.5, the prepared porous carbon fibres exhibited a high SSA of 918 m^2^ g^−1^ in 3 mol L^−1^ KOH aqueous electrolyte. The unique pore structure of this porous carbon fibre made it a suitable substrate material for high-performance electrodes. The large number of pores provided a rich active surface for the attachment of high-quality pseudocapacitive active materials. The continuous carbon skeleton provided fast conduction channels for electrons. The interconnected pores facilitated the accelerated conduction of ions within the pores. In addition to PAN-b-PMMA for the preparation of porous carbon, block copolymers such as PS-b-PEO, PAN-b-PBA, PS-b-PAN and other block copolymers have also been studied by researchers [99,100,101]. The SSA of most porous carbons prepared from these block copolymers is still less than 1500 m^2^ g^−1^. The ultimate SSA of porous carbons prepared from block copolymers needs to be further increased by controlled molecular design or other methods.

The preparation of copolymer precursors takes a long time and requires a tedious procedure. Copolymer precursors are also expensive and non-renewable. Among the emerging methods, the preparation of porous carbons by the self-activation of carbohydrates is now also in the public eye. Carbohydrates are abundant in nature and the preparation of porous carbons directly from carbohydrates reduces the use of activators. Zhang et al. investigated the production of porous carbon with ultra-high SSA and controlled pore structure through hydrogel-controlled carbonisation and self-activation processes of glucose (Figure 5c,d) [102]. Polyacrylamide was used as a pore-forming agent and the pore structure could be adjusted by controlling the polyacrylamide content. At a polyacrylamide content of 60%, the highest SSA of porous carbon could reach 3381 m^2^ g^−1^. In addition to this, some of the carbohydrates can be prepared as porous carbon by a hydrothermally assisted carbonisation process or by direct pyrolysis [103,104]. Due to the uniqueness of the carbohydrate self-activation mechanism, more oxygen-rich organic precursors can be used for the synthesis of porous carbons.

There is also a more specific method of preparing porous carbons, which is a new technique based on photochemical and photothermal reactions between the laser spot and the carbon precursor. First proposed by Ajayan et al. in 2011, reduced graphene oxide with a 3D morphology, high capacitance and high electrical conductivity was prepared [105]. Xu et al. prepared a honeycomb porous graphene material using a laser-scribing technique (Figure 5e–i) [106]. A single layer of porous graphene with a thickness of 48.3 µm, this material has the advantage of being low-cost and suitable for large-scale manufacture, and is particularly suitable for monitoring weak physiological signals such as the human pulse, breathing and throat movements. Given the advantages of laser scribing, there has been an increasing number of researchers developing porous graphene electrodes with high area capacitance and high rate capability, and this work is worth looking forward to [107,108]. In addition to the three emerging methods, there are also experimental methods such as self-grown nanomaterials on flexible conductive substrates to prepare porous carbons [109], which will not be repeated in this review.

**Table 3:** Comparison of porous carbons prepared by other methods.

Methods	Basic Steps (Raw Hole Mechanism)	Advantages	Disadvantages	Ref.
Direct pyrolysis of block copolymers method	Decomposition of polymers or functional groups	No activation step required	Expensive and non-renewable copolymer precursors	[98]
Carbohydrate self-activation method	CO_2_ + C → 2CO H_2_O + C → CO + H_2_	High availability in nature	Expensive organic precursors	[102]
Laser-scribing method	CO_2_ laser promotes thermal expansion to create pores	No template required + No activation step required	Less control over pore size	[106]
Flexible substrate self-growth method	3D metal oxide nanoplates synthesised on highly hydrophobic carbon cloth substrates	Greener, easier and more scalable	___	[109]

In summary, the synthesis of porous carbons is becoming more and more diverse, from the traditional carbonisation-activation method to the template method and other emerging methods, with a preference for low energy consumption, low cost and low pollution. The methods that exist in the laboratory for preparing porous carbons will gradually move towards industrial production, which is something to look forward to.

## 3. Application of Porous Carbons as Supercapacitor Electrodes

Some methods for synthesis of porous carbons have been described previously, and porous carbons will obtain further applications. This is because one of the ultimate goals of supercapacitor research is to achieve high charge-storage capacity at ultra-high scan rates or current densities. Carbon, with its high electrical conductivity, is the ideal material to achieve this goal. Smaller resistances enable fast electron transfer, which is essential for the high-rate performance of supercapacitors. However, the specific capacitance of carbon has remained at a moderate level between 100 and 200 F g^−1^ for decades. Previous studies have found that the relatively small ion-accessible area of carbon materials is the main reason for their limited capacitance [50,77]. The porous carbons vary in pore size and are broadly classified into four types, namely, microporous, mesoporous, macroporous and hierarchically porous. This is the 1985 IUPAC classification of pores based on pore width. Pore width is defined as the pore diameter, or specifically the fissure-like pore (i.e., the void between two layers), and the distance between layers [110]. This review divides porous carbons as supercapacitor electrodes in recent years into non-heteroatom-doped and heteroatom-doped types. Within each type the classification is described in terms of the different pore sizes of the porous carbons.

### 3.1. Heteroatom-Free Doped Porous Carbons

This section focuses on the application of heteroatom-free doped porous carbons as supercapacitor electrodes, as listed in Table 4, which will be described in detail below.

#### 3.1.1. Microporous Carbons

**Figure 6:**
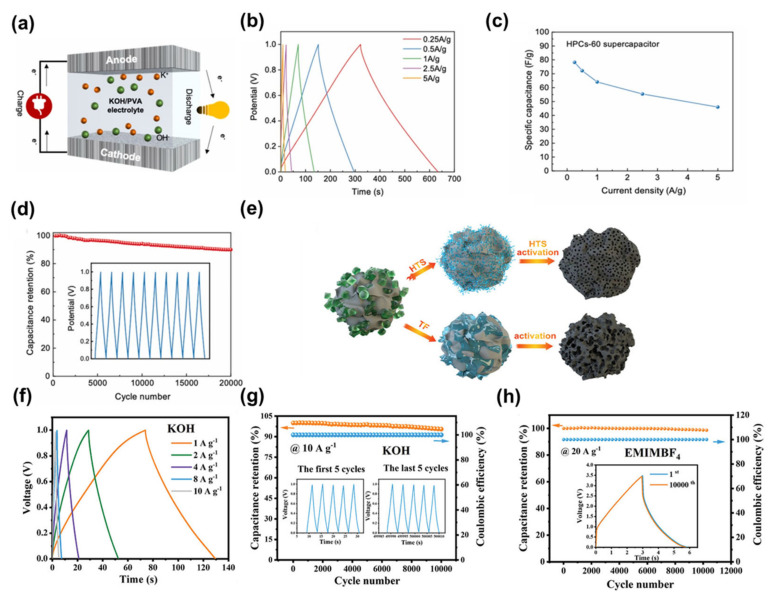
(**a**) Schematic diagram of a quasi-solid-state supercapacitor. (**b**) GCD curves of supercapacitors at different current densities. (**c**) Specific capacitance of supercapacitors at different current densities. (**d**) Long-cycle performance of supercapacitors. Reproduced with permission from Ref. [102]. Copyright 2023 Elsevier Ltd. (**e**) Schematic diagram of the mechanism of porous carbon formation by HTS-KOH activation and conventional tube furnace-KOH activation. (**f**) GCD curves at 1–10 A g^−1^. (**g**) Long cycle performance of supercapacitors with 6 M KOH as the electrolyte at 10 A g^−1^. (**h**) Long cycle performance of supercapacitors with EMIMBF_4_ ionic liquid as the electrolyte at 20 A g^−1^. Reproduced with permission from Ref. [111]. Copyright 2022 Wiley-VCH.

Zhang et al. used polyacrylamide as a microporous pore generator, where the pore structure can be adjusted by controlling the polyacrylamide content [102]. After testing, they found that the SSA of the porous carbon reached 3381 m^2^ g^−1^ at 60% polyacrylamide content. They then used this porous carbon electrode with a KOH/PVA gel electrolyte to prepare a quasi-solid-state supercapacitor (Figure 6a). The supercapacitor exhibited a perfectly symmetrical triangular Galvanostatic charge/discharge (GCD) curve (Figure 6b). The specific capacitance of the device was calculated on the basis of the GCD test. A capacitance of 78.2 F g^−1^ was achieved at a current density of 0.25 A g^−1^, with 59% capacitance retention at a high current density of 5 A g^−1^ (Figure 6c). In addition, cyclic stability is a key factor that must be considered in practical applications. The authors achieved a high capacitance retention of 89.9% for the device after 20,000 charge/discharge cycles at a current density of 10 A g^−1^, and the GCD curve remained symmetrically shaped at the end of the test (Figure 6d). As another example, Chen et al. proposed a method of carbonisation and KOH activation by rapid high-temperature impact [111]. The instantaneous melting of KOH into small droplets under the impact of rapid high temperatures can effectively produce a large number of pores of uniform size distribution, which is conducive to the interaction between carbon and KOH to form controlled and dense micropores (Figure 6e). The specific capacitance of this porous carbon-based supercapacitor was 28.1, 24, 22, 15.2 and 13 F g^−1^ at current densities of 1, 2, 4, 8 and 10 A g^−1^ in a 6 M KOH electrolyte, respectively (Figure 6f). It also exhibited remarkable cycling stability, with 95.53% capacitance retention and 100% coulombic efficiency even after 10,000 cycles at a current density of 10 A g^−1^ (Figure 6g). After the electrolyte was replaced with a 1-Ethyl-3-methylimidazolium tetrafluoroborate (EMIMBF_4_) ionic liquid electrolyte, the supercapacitor exhibited a capacitance retention of 98.64%, a coulombic efficiency of 100% and good cycling stability after 10,000 cycles at a current density of 20 A g^−1^ (Figure 6h). 

#### 3.1.2. Mesoporous Carbons

**Figure 7:**
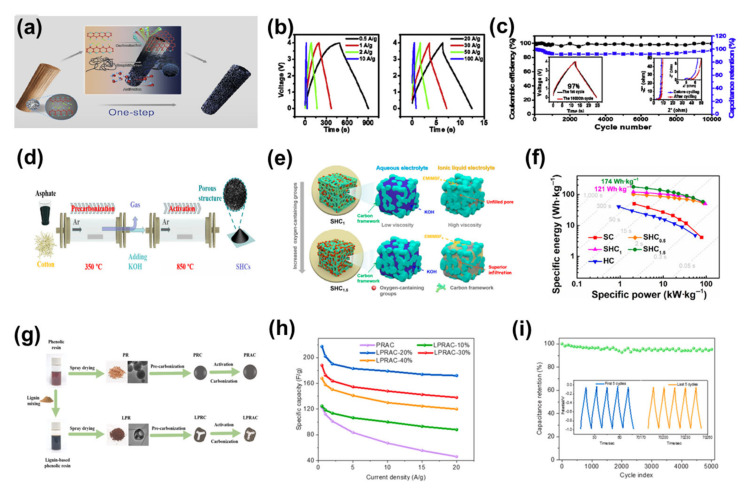
(**a**) Schematic diagram of the synthesis of mesoporous activated carbon fibres. (**b**) GCD curves of mesoporous activated carbon fibres at different current densities. (**c**) Long cycle performance of supercapacitors based on mesoporous activated carbon fibres, with inset GCD curves for the 1st and 10,000th cycles and EIS curves before and after cycling. Reproduced with permission from Ref. [113] Copyright 2019 Elsevier Ltd. (**d**) Schematic diagram of the soft and hard carbon synthesis schematic. (**e**) Visible distribution of the two electrolytes within the different hard and soft carbon pores. (**f**) Ragone diagram of SHC-based symmetric supercapacitor. Reproduced with permission from Ref. [114] Copyright T singhua University Press and Springer-Verlag GmbH Germany, part of Springer Nature 2021. (**g**) Schematic diagram of porous carbon synthesised by spray drying process. (**h**) Specific capacitance of supercapacitors at different current densities. (**i**) Long cycle performance of supercapacitors with LPRAC-20% electrodes at 20 A g^−1^. Reproduced with permission from Ref. [120] Copyright 2022 Elsevier Ltd.

Many scholars have studied mesoporous carbon nanofibers, such as Qian et al. who prepared highly conductive mesoporous activated carbon fibres by CO_2_ controlled carbonisation and activation of polyacrylonitrile-based fibres at high temperatures (Figure 7a), which can be used for 4V supercapacitors in ionic liquids [112,113]. The mesoporous activated carbon fibre exhibited an extremely high SSA (2404 m^2^ g^−1^), large mesopore volume (2.3 cm^3^ g^−1^), a large filling density (0.25 g cm^−3^), and good chemical stability at high voltages with a conductivity of 57–195 S cm^−1^. Testing the electrolyte at 4 V in EMIMBF_4_, the mesoporous activated carbon fibres demonstrated a high capacitance (204 F g^−1^ at 0.5 A g^−1^), high energy density (113 Wh kg^−1^) and excellent long cycle performance (Figure 7b,c). The excellent capacitive properties were attributed to the one-dimensional (1D) structure of the mesoporous activated carbon fibres, which had a long in-plane length in the electron transfer axis and a short radial diffusion distance for the ions of the ionic liquid. The mesoporous activated carbon fibres obtained in this study are the first to combine all the advantages of traditional electrode materials (activated carbon) and new generation of electrode materials (mainly carbon nanotubes and graphene) and to minimise their main disadvantages. In another example, Qiu et al. implanted long-range ordered structures in situ into an amorphous bio-based carbon, using the inherent properties of petroleum pitch liquid phase carbonisation, and proposed a petroleum pitch mediated co-conversion technique to modify the solid phase conversion/carbonisation process of biomass (Figure 7d), resulting in the construction of mesoporous soft and hard carbon materials with long- and short-coupled ordered structures on a small scale [114]. The SSA of the porous carbon obtained was greater than 2000 m^2^ g^−1^. As a result, the specific capacitance of the supercapacitor in 6 M KOH reached 387 F g^−1^ at a current density of 1 A g^−1^. In addition, the high mesopore ratio of around 60% facilitated its application in highly viscous ionic liquid electrolytes. The experimental results showed that the supercapacitor with EMIMBF_4_ as the electrolyte exhibited a high specific energy of 174 Wh kg^−1^ at 2 kW kg^−1^ and still maintained a high specific power of 50 kW kg^−1^ at 78 Wh kg^−1^ (Figure 7e,f) [115,116,117,118,119]. There are also researchers such as Si et al. who directly blended lignin of different mass ratios (10%, 20%, 30%, 40%) with phenolic resin and dried the materials by spray drying [120]. The resulting phenolic resin was then pre-carbonised and KOH activated with a lignin-based phenolic resin to give a ‘caged’ lignin-based phenolic resin mesoporous carbon material (LPRAC) (Figure 7g). The authors used these mesoporous carbons for supercapacitor electrode materials at a current density of 0.5 A g^−1^, with specific capacitances of 124.5, 217.3, 188.0 and 167.8 F g^−1^ for the LPRAC-10%, LPRAC-20%, LPRAC-30% and LPRAC-40% electrodes, respectively. The specific capacitance of the LPRAC-10%, LPRAC-20%, LPRAC-30% and LPRAC-40% electrodes decreased to 88.0, 172.5, 138.4 and 120.1 F g^−1^, respectively, when the current density was increased to 20 A g^−1^. The corresponding capacitance retention rates were 37.5%, 70.5%, 79.2%, 73.4% and 71.5%, respectively (Figure 7h). It can be seen that the capacitance retention of the LPRAC-20% electrode is better than the other samples. Excessive lignin incorporation can lead to a reduction in the specific capacitance of porous carbon materials. This phenomenon may be related to the negative effect of excess lignin on the pore formation of LPRAC-*x*. In addition, the cycling stability of the supercapacitor is crucial. The cycling stability was assessed by performing a GCD test on the device at a current density of 20 A g^−1^ for 5000 cycles (Figure 7i). After 5000 consecutive cycles, the capacity retention rate reached 95.34%, indicating that the supercapacitor had good cycling stability.

#### 3.1.3. Macroporous Carbons

**Figure 8:**
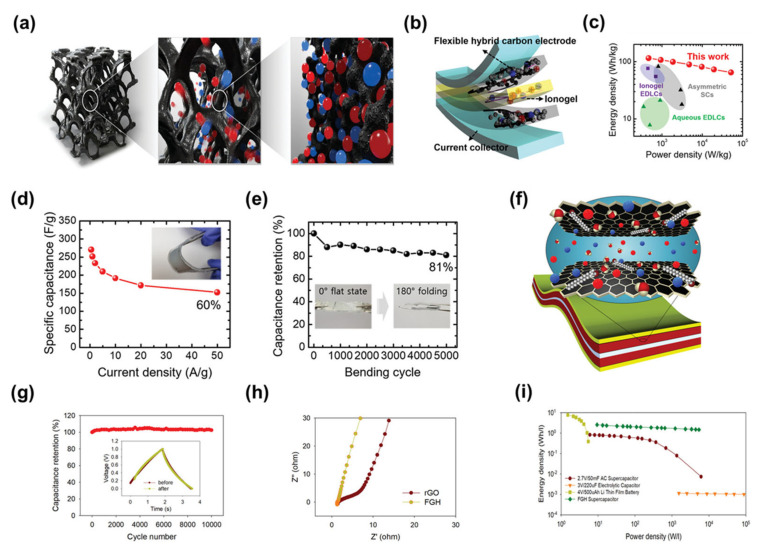
(**a**) Schematic of three-dimensional macro/mesoporous carbon (**left**), internal ion transport facilitation (**middle**), and capacitive energy storage on porous surface (**right**). (**b**) Schematic of all-solid-state symmetric supercapacitor. (**c**) Ragone plot of flexible supercapacitor. (**d**) Variation of specific capacitance at different current densities. (**e**) Capacitance of flexible supercapacitor after 5000 consecutive folding cycles between 0° and 180° retention. Reproduced with permission from Ref. [121] Copyright 2020 WILEY-VCH. (**f**) Flexible supercapacitors based on macroporous graphene electrodes. (**g**) Long cycle performance of supercapacitors. (**h**) Electrochemical impedance spectroscopy of the supercapacitors. (**i**) Comparison of different supercapacitor energy densities and power densities with literature reports and some commercial energy storage devices. Reproduced with permission from Ref. [127] Copyright 2022 Wiley-VCH.

In most cases, macroporous carbon materials inevitably contain micropores/mesopores [44]. Yoo et al. creatively introduced macroporous carbon electrodes into supercapacitors to address the problem that when ionic gels are used as electrolytes in supercapacitors, their large, slow-moving ions cannot effectively enter the pores of conventional microporous carbons [121]. They reported a strategy to optimise the electrochemically active surface of carbon electrodes and thereby improved energy storage performance by combining meso/macroporous carbon with ionic gel electrolytes. This design facilitated the mass transport of electrolyte ions in the solid ionic gel electrolyte and effectively utilised the carbon electrode surface for capacitive energy storage, resulting in a high energy storage performance that exceeded the Ragone limit. An all-solid-state supercapacitor with excellent bending/folding durability was also successfully demonstrated (Figure 8a,b). When the current density was 0.5 A g^−1^, the energy density was up to 115 Wh kg^−1^ and the power density was 467 W kg^−1^; when the current density was 50 A g^−1^ and the energy density was 65 Wh kg^−1^, the power density reached up to 51,771 W kg^−1^ (Figure 8c) [122,123,124,125,126]. With a current density of 1 A g^−1^ to 50 A g^−1^, the capacity retention of the supercapacitor also remained at 60% (Figure 8d). The all-solid-state supercapacitor also had good bending capability, with 81% capacity retention after 5000 repetitions of the 0°–180° bending test (Figure 8e). In another example, B. Kaner et al. proposed a simple electrochemical method for the direct deposition of functionalised graphene frameworks using electrostatic interactions between graphene oxide and cationic surfactants [127]. This work provided a simple and effective strategy for the preparation of macroporous carbon electrodes (Figure 8f). They used the macroporous carbon as an electrode in a supercapacitor to verify its electrochemical performance. During the first few hundred cycles, the capacitance of the device increased slightly, probably due to further activation of the electrodes. This was followed by a stable capacitance with essentially no losses over nearly 10,000 cycles, demonstrating the excellent long-cycle stability of the supercapacitor (Figure 8g). It can also be found that the supercapacitor has a fast electrochemical response by electrochemical impedance spectroscopy. Better performance than graphene supercapacitors was made by chemically reduced rGO. The supercapacitor also had a surface energy density of 19.1 µWh cm^−2^ at a power density of 68 µW cm^−2^. Even at an ultra-high-power density of 39,674 µW cm^−2^, the supercapacitor maintained an energy density of more than 11 µWh cm^−2^, which is an advantage over various graphene-based supercapacitors. Its performance is also superior to that of the recently reported graphene-based supercapacitors (Figure 8h,i) [128,129,130,131].

#### 3.1.4. Hierarchically Porous Carbons

3D-layered porous carbon materials containing micropores, mesopores and macropores are in demand for multifunctional applications as they combine the advantages of uniquely functional microporous, mesoporous and macroporous channels allowing exposure of abundant active sites (micropores) with rapid diffusion (mesopores/macropores) [132].

**Figure 9:**
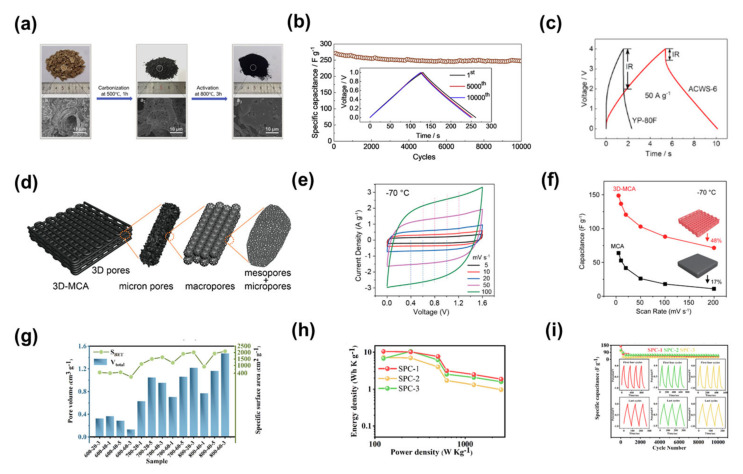
(**a**) Schematic diagram of the preparation of walnut-shell-based activated carbon. (**b**) Long cycle performance of supercapacitors based on walnut-shell-based activated carbon electrodes. (**c**) GCD profiles at 50 A g^−1^. Reproduced with permission from Ref. [133]. Copyright 2020 Elsevier Ltd. (**d**) Schematic diagram of 3D-printed porous carbon aerogels with different pores. (**e**) CV curves of porous carbon aerogels at different scan rates at −70 °C. (**f**) Variation of the specific capacitance of 3D porous carbon aerogels and normal porous carbon aerogels with scan rate at −70 °C. Reproduced with permission from Ref. [144]. Copyright 2021 American Chemical Society. (**g**) Bioactivity characteristics of Sargassum carbon activity characteristics. (**h**) Ragone plots of Sargassum biochar with different pore sizes. (**i**) Long cycle performance of Sargassum biochar with different pore sizes. Reproduced with permission from Ref. [146]. Copyright 2022 Elsevier Ltd.

Yang et al. reported on sheet porous carbon prepared by carbonisation and activation of walnut shells for use in high power supercapacitors (Figure 9a) [133]. The walls of its pores consist of 1–2 carbon layers. This walnut-shell-based activated carbon has a high SSA of up to 3577 m^2^ g^−1^. The supercapacitor retained 95% of its initial capacitance after 10,000 cycles at a current density of 1 A g^−1^. At 50 A g^−1^, the material also has a low electrical resistance (as shown by the red curve) (Figure 9b,c). Its supercapacitor energy density and power density are higher than those previously reported for biomass-derived carbon materials and partially graphene-based porous materials [115,134,135,136,137,138,139,140,141,142,143]. Another example of supercapacitor performance at low temperatures was investigated by researchers such as Li et al. who fabricated 3D-printed 3D porous carbon aerogels through a unique combination of chemical methods and direct ink writing techniques (Figure 9d) [144]. The 3D porous carbon aerogel had an open porous structure with a large SSA of approximately 1750 m^2^ g^−1^, and the CV curves of the 3D porous carbon aerogel at −70 °C showed a box shape at different scan rates (Figure 9e). It revealed that ions and electrons diffused rapidly at −70 °C. When the scan rate was increased from 5 to 200 mV s^−1^, the specific capacitance of the 3D porous carbon aerogel electrode dropped from 148.6 to 71.4 F g^−1^, with a capacitance retention rate of 48%. In contrast, the ordinary porous carbon aerogel had a capacitance retention rate of only 17% (Figure 9f). These impressive results and the related studies highlight the important role of the open porous structure in maintaining the capacitive performance of supercapacitors at ultra-low temperatures [145]. There are also researchers such as Sun et al., who investigated the relationship between the structural characteristics of biomass porous carbon and the electrochemical performance of supercapacitors in order to find a valid correlation between the preparation process of symmetric supercapacitors and their application performance [146]. Based on the responsive experimental design, the pore structure of Sargassum bio-porous carbon (SPC) was adjusted by the synergistic optimisation of key parameters and the hierarchical Sargassum porous carbon with different pore characteristics was successfully prepared by microwave-parameter pyrolysis. The SSA of the hierarchical Sargassum porous carbon prepared at different microwave parameters ranged from 113.06–2103.20 m^2^ g^−1^ and the pore volume from 0.1376–1.4756 cm^3^ g^−1^ (Figure 9g). They selected three hierarchical Sargassum porous carbons with different pores for use in supercapacitor electrodes. At a current density of 1 A g^−1^, the SPC-1 power density was 125 W kg^−1^. The maximum energy density at this point was 10.47, 6.75 and 7.26 Wh kg^−1^ for SPC-1, SPC-2 and SPC-3, respectively (Figure 9h). The energy density of devices based on SPC-1 electrodes was higher than other electrodes due to the rational pore structure of the carbon electrode which had a better effect on ion storage and transport in the electrolyte. The supercapacitors prepared using these three electrode materials maintained 78.6%, 92.7% and 91.2% capacity after 10,000 cycles at a current density of 1A g^−1^ for SPC-1, SPC-2 and SPC-3, respectively (Figure 9i). The results showed that the pore structure of the porous carbon remained stable during the cyclic charging and discharging process. The above tests can provide technical support and a theoretical basis for the control of the pore network and the targeted optimisation of the supercapacitor performance by adjusting pore structure of the biochar. 

**Figure 10:**
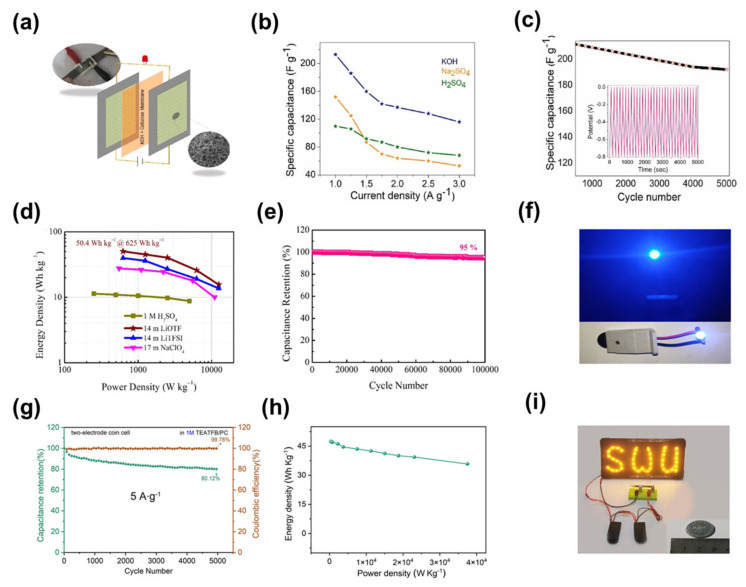
(**a**) Fabrication of porous carbon symmetric electrode. (**b**) Specific capacitance values at various current densities based on porous activated carbon. (**c**) Long-term cycle performance of supercapacitors. Reproduced with permission from Ref. [147] Copyright 2022 Elsevier Ltd. (**d**) Ragone plots of supercapacitors based on hierarchically porous carbon nanosheets using different electrolytes. (**e**) Long-term cycle performance of supercapacitors with 14 M LIOTF as electrolytes at a current density of 10 A g^−1^. (**f**) Optical image of a supercapacitor based on layered porous carbon nanosheet electrodes to light up an LED lamp. Reproduced with permission from Ref. [148]. Copyright 2022 American Chemical Society. (**g**) Long-term cycle performance of supercapacitors based on hypercrosslinked polymer-based porous carbon electrodes at a current density of 5 A g^−1^. (**h**) Ragone plots of porous carbon electrodes based on hypercrosslinked polymers. (**i**) Photos of 8 samples of supercapacitors connected in series and parallel to power a yellow-orange LED. Reproduced with permission from Ref. [149]. Copyright 2023 Elsevier B.V.

Ajayan et al. chose Tasmanian blue gum bark as the raw material for the preparation of activated porous carbon, and the SSA of the obtained porous carbon was 971 m^2^ g^−1^ [147]. They fabricated a symmetric supercapacitor based on this porous carbon electrode (Figure 10a), which exhibited a high electrochemical storage capacity of 212 F g^−1^ at a current density of 1 A g^−1^ using 1 M KOH as the electrolyte. After 5000 charge-discharge cycles, the capacity retention rate of the supercapacitor was 89%, indicating that it had good long-term cycle stability (Figure 10b,c). In another example, Liu et al. used the pore-enlarging effect of trimethylbenzene, and hydrophobic trimethylbenzene-mediated interfacial assembly with carbon precursors and pore segments through π−π bonds and van der Waals interactions in different volumes of solvents to generate well-dispersed micelles, which could form tunable mesopores on hierarchically porous carbon under certain conditions [148]. Their symmetric supercapacitor, assembled based on this porous carbon, exhibited an energy density of 50.4 Wh kg^−1^ at a power density of 625 W kg^−1^ when using 14 M LiOTF electrolytes. The energy density was 39.8 Wh kg^−1^ when the power density was 625 W kg^−1^ using 14 M LiTFSI electrolytes. The energy density was 27.7 Wh kg^−1^ when the power density was 550 W kg^−1^ using 17 M NaClO_4_ electrolytes. The energy density was 11.3 Wh kg^−1^ when the power density was 250 W kg^−1^ using 1 M H_2_SO_4_ electrolyte (Figure 10d). In addition, the device also had a good long-term cycle capability. At a current density of 10 A g^−1^, when 14 M LiOTF was used as the electrolyte, the device had a capacity retention rate of 95% after 10,000 charge-discharge cycles (Figure 10e). in practical applications, the device was able to make an LED lamp work and maintain a good working condition (Figure 10f). Also, Chen et al. made a hierarchically porous carbon by activating carbonised polymer dots and hypercrosslinked polymer composites, and the obtained porous carbon had an ultrahigh SSA of 4241 m^2^ g^−1^ [149]. The supercapacitor prepared based on the porous carbon had a capacity retention rate of 80.12% after 5000 charge-discharge cycles at a high current density of 5 A g^−1^, indicating that the device had a good long-term cycle capability (Figure 10g). From the Ragone diagram, it can be seen that the energy density of the device could reach 47 Wh kg^−1^ when the power density was 750 W kg^−1^, and the energy density still maintained 35.8 Wh kg^−1^ even when the power density was 37,497 W kg^−1^ (Figure 10h) [150,151,152,153,154,155,156]. The authors also applied the device in practical work, and the device was able to light up the LED lamp stably for 3 min without turning off (Figure 10i). Unless otherwise specified, the devices assembled with hierarchically porous carbons as supercapacitor electrodes in the above examples are electric double layer-dominated supercapacitors.

**Table 4:** Typical applications of different types of heteroatom-free porous carbons.

Type	Electrolyte	2 Electrode/ 3 Electrode System	Average Pore Size (nm)	Pore Volume (cm^3^ g^−1^)	SSA (m^2^ g^−1^)	Ref.
Microporous carbon	KOH/PVA	2 electrodes	1.26	—	3381	[102]
	6 M KOH, EMIMBF_4_	2 electrodes	1.75	—	843.15	[111]
Mesoporous carbon	EMIMBF_4_	2 electrodes	6.6	—	2404	[113]
	EMIMBF_4_	2 electrodes	2–4	—	2837	[114]
	6 M KOH	3 electrodes	2.23	1.059	1899.5	[120]
Macroporous Carbon	[EMI][BF4] ionogel	2 electrodes	127	2.9	880	[121]
	1.0 M H_2_SO_4_	2 electrodes	—	—	175.3	[127]
Hierarchically Porous Carbon	6 M KOH, EMIMBF4	2 electrodes	3.31	2.19	3577	[133]
	0.5 M tetraethylammonium tetrafluoroborate (TEABF_4_)	2 electrodes	2–300	—	1750	[144]
	1 M Na_2_SO_4_	2 electrodes	2–4	1.476	2103.2	[146]
	1 M KOH, 1 M Na_2_SO_4_, 1 M H_2_SO_4_	3 electrodes	1.5–3.9	0.26	971	[147]
	1 M H_2_SO_4_	3 electrodes	2–3.5	1.31	2233	[148]
	1 M TEATFB/PC	2 electrodes	2.14	2.5	4241	[149]

### 3.2. Heteroatom-Doped Porous Carbons

This section focuses on the application of heteroatom-doped porous carbons as supercapacitor electrodes, as shown in Table 5, which will be described in detail below.

#### 3.2.1. Heteroatom-Doped Microporous Carbons

**Figure 11:**
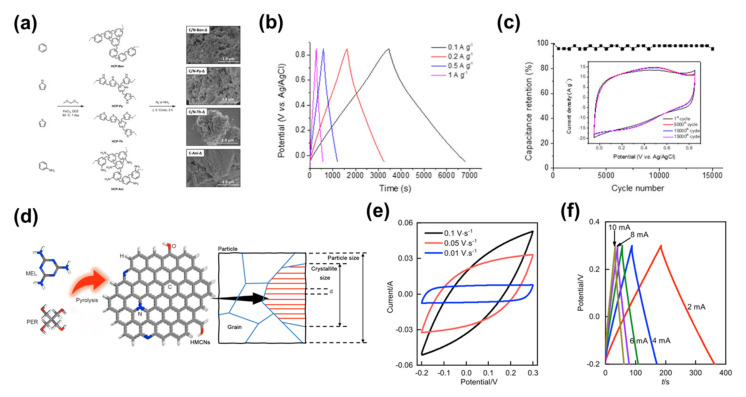
(**a**) Synthesis of supercross-linked polymers and their carbonisation method. (**b**) GCD curves of heteroatom-doped microporous carbon-based supercapacitors at 0.1–1 A g^−1^ current density. (**c**) Long cycle performance of the heteroatom-doped microporous carbon-based supercapacitors at a current density of 5 A g^−1^. Reproduced with permission from Ref. [157]. Copyright: this is an open access article under the CC BY license. (**d**) Schematic diagram of the structure of heteroatom-doped microporous carbon nanosheets. (**e**,**f**) CV and GCD curves of supercapacitors based on heteroatom-doped microporous carbon nanosheet electrodes. Reproduced with permission from Ref. [158]. Copyright 2022 Elsevier Ltd.

Cooper et al. studied an important class of porous materials, super-crosslinked polymers [157]. These materials can be synthesised from aromatic precursors using a one-step ‘weaving’ procedure (Figure 11a). They have great potential for synthetic diversification. However, the lack of electrical conductivity of the original super-crosslinked polymers limits their application in electrochemistry. Doping heteroatoms by selecting raw materials and varying the type of gas used in the carbonisation can obtain heteroatom-doped microporous carbons with the desired supercapacitor performance. The GCD curves for the supercapacitors based on this heteroatom-doped microporous carbon all showed a symmetrical triangular distribution between 0.1 and 1 A g^−1^ current densities (Figure 11b), indicating the typical super-capacitive behaviour of this material. The material had an extremely high capacitance of 374 F g^−1^ at a current density of 0.1 A g^−1^, and revealed better electrode performance than many other heteroatom-doped carbon materials. The supercapacitor also exhibited a good long cycle capability, with a capacitance retention of 95.8% after 15,000 charge/discharge cycles at a current density of 5 A g^−1^ (Figure 11c). In another example, Liu et al. demonstrated a solvent-free, template-free, one-pot condensation method for the synthesis of heteroatom-doped microporous carbon nanosheets using melamine (MEL) and pentaerythritol (PER) as precursors [158]. By varying the ratio of melamine to pentaerythritol and the pyrolysis temperature, the amount of doping, surface area and porosity of the heteroatom-doped microporous carbon nanosheets can be controlled (Figure 11d). They prepared the best heteroatom-doped microporous carbon nanosheets with high N and O doping contents (10.7 and 6.5 wt%, respectively), high SSA (539 m^2^ g^−1^), high microporosity (0.18 m^3^ g^−1^), nanosheet morphology and abundant surface functional groups. Due to these remarkable properties, the CV of the supercapacitors based on this electrode was stable at different scan rates and had a high specific capacitance (475 F g^−1^ at a current density of 1.3 A g^−1^) and fast charge and discharge rates (Figure 11e,f). This research presents a novel, resource efficient and environmentally friendly approach to heteroatom-doped microporous carbon nanosheets for energy and environmental applications. 

#### 3.2.2. Heteroatom-Doped Mesoporous Carbons

**Figure 12:**
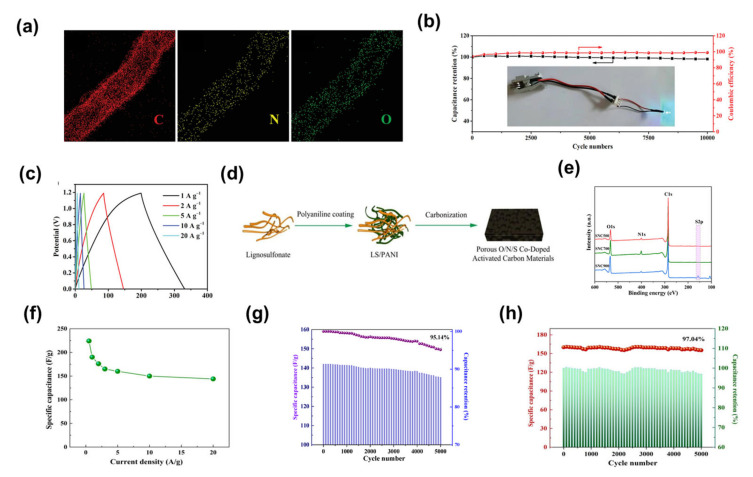
(**a**) EPS elemental mapping of the N-doped mesoporous carbon nanotubes. (**b**) Long cycle performance of supercapacitors based on the N-doped mesoporous carbon nanotube electrodes. (**c**) GCD curves at different current densities. Reproduced with permission from Ref. [69]. Copyright 2022 Wiley-VCH. (**d**) Schematic of oxygen-nitrogen-sulfur co-doped active mesoporous carbon. (**e**) XPS spectra of the oxynitride-sulfur co-doped active mesoporous carbon. (**f**) Specific capacitance of the oxynitride-sulfur co-doped active mesoporous carbon-based supercapacitors at different current densities. (**g**) Specific capacitance and capacitance retention rate of electrode material after 5000 cycles. (**h**) Long cycle performance of the oxynitride-sulfur co-doped active mesoporous carbon-based supercapacitors. Reproduced with permission from Ref. [162]. Copyright 2022 by the authors.

Wang et al. prepared nitrogen-doped mesoporous carbon nanotubes by the hard-template method [69]. The resulting nitrogen-doped mesoporous carbon nanotubes had a large SSA (1037 m^2^ g^−1^) with uniform nitrogen doping (Figure 12a). The supercapacitor based on nitrogen-doped mesoporous carbon nanotube electrodes showed a capacitance retention of 98.2% after 10,000 charge/discharge cycles at a current density of 10 A g^−1^, and the Coulombic efficiency remained almost 100% (Figure 12b). This indicates that the device had very good long-cycle performance. It had an energy density of 11.6–8.8 Wh kg^−1^ and an increased power density from 313 to 6261 W kg^−1^, which is better than most reported porous carbon-based symmetrical supercapacitors [111,159,160,161]. In addition, the shape of the charge-discharge curves of the device at different current densities is similar, which also demonstrates the stability of the device (Figure 12c). In another example Wu et al. used a two-step calcination method to prepare oxygen, nitrogen and sulphur co-doped reactive mesoporous carbon with uniform oxygen, nitrogen and sulphur doping (Figure 12d,e) [162]. Supercapacitors based on oxygen, nitrogen and sulphur co-doped active mesoporous carbon electrodes demonstrated good cycling performance. As the current density varied from 0.5, 1, 2, 3, 5, 10 and 20 A g^−1^, the corresponding specific capacitance reached 224.2, 190.1, 176.2, 165.5, 159.8, 150.1 and 144.0 F g^−1^, respectively (Figure 12f). At a current density of 5 A g^−1^, the single-electrode and assembled supercapacitors achieved a capacity retention of 95.14% and 97.04%, respectively, after 5000 charge-discharge cycles (Figure 12g,h). In addition, the assembled device had a high energy density of 8.33 Wh kg^−1^ at a power density of 62.5 W kg^−1^. At an ultra-high-power density of 5000 W kg^−1^, the energy density remained at 5.01 Wh kg^−1^, which is better than that previously reported in the literature [163,164]. This study provides an efficient method for the preparation of oxygen-nitrogen-sulphur co-doped active mesoporous carbons and their application to high-performance supercapacitors. 

#### 3.2.3. Heteroatom-Doped Macroporous Carbons

**Figure 13:**
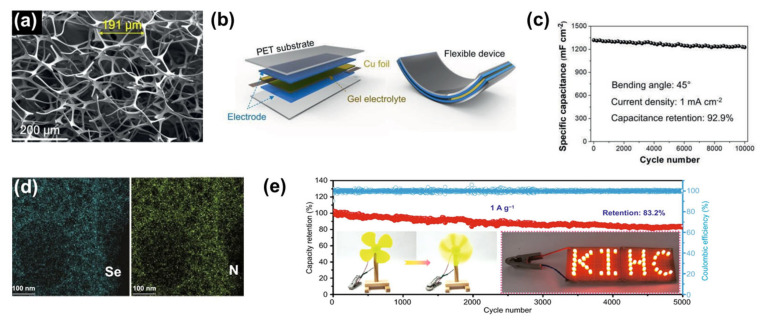
(**a**) SEM image of the 3D N-doped carbon nanotubes. (**b**) Schematic diagram of a flexible supercapacitor based on the 3D N-doped carbon nanotubes. (**c**) Long-cycle performance of a flexible supercapacitor based on the 3D N-doped carbon nanotubes. Reproduced with permission from Ref. [165]. Copyright 2020 Wiley-VCH. (**d**) EDS elemental mapping of Se and N in the Se/N co-doped 3D macroporous carbon. (**e**) Long-cycle performance of supercapacitors based on the Se/N co-doped 3D macroporous carbon. Reproduced with permission from Ref. [166]. Copyright The Author(s) 2021.

Terrones et al. prepared 3D nitrogen-doped carbon nanotube electrode materials using melamine foam as a raw material [165]. Nickel ferrate/nitrogen-doped carbon nanotube composite electrode materials were also synthesised by simple ion adsorption, vapour phase diffusion-deposition and heat treatment. Nickel ferrate/nitrogen-doped carbon nanotube composite electrode materials possessed an excellent macroporous structure (Figure 13a), and were used to assemble a flexible supercapacitor with polyethylene terephthalate as the substrate and PVA/KOH gel as the electrolyte (Figure 13b). The high nitrogen content in melamine enabled the doping of the carbon skeleton with nitrogen atoms, giving the electrode material better electrochemical activity. In addition, the 3D open macroporous structure provided ideal conditions for the homogeneous loading of nickel ferrate. The apparent density of the resulting nickel ferrate/nitrogen-doped carbon nanotube foam-composite electrode material was only 0.0055 g cm^−3^. Thanks to the open macroporous structure of the carbon skeleton and the synergy between nickel ferrate and nitrogen-doped carbon nanotubes, the supercapacitor revealed a good long-cycle performance, with 92.9% capacitance retention after 10,000 cycles at a surface current density of 1 mA cm^−2^ (Figure 13c). In another example Hu et al. developed a simple and versatile self-sacrificing template method to prepare Se/N co-doped 3D macroporous carbon with an extended interlayer structure and abundant active sites and uniform doping of Se and N on the 3D macroporous carbon (Figure 13d) [166]. The resulting macroporous carbon doped with heteroatoms was used as the electrode for the hybrid supercapacitor, and the assembled supercapacitor has a good long-cycle performance. At a current density of 1 A g^−1^, 83.2% of capacity was maintained after 5000 cycles (Figure 13e). This work opens up new avenues for the development and application of high-performance hybrid capacitors.

#### 3.2.4. Heteroatom-Doped Hierarchically Porous Carbons

Like heteroatom-doped porous carbons, heteroatom-doped hierarchically porous carbon has unique properties compared with heteroatom-doped microporous carbons, heteroatom-doped mesoporous carbons, and heteroatom-doped macroporous carbons, said to be more popular with scholars.

**Figure 14:**
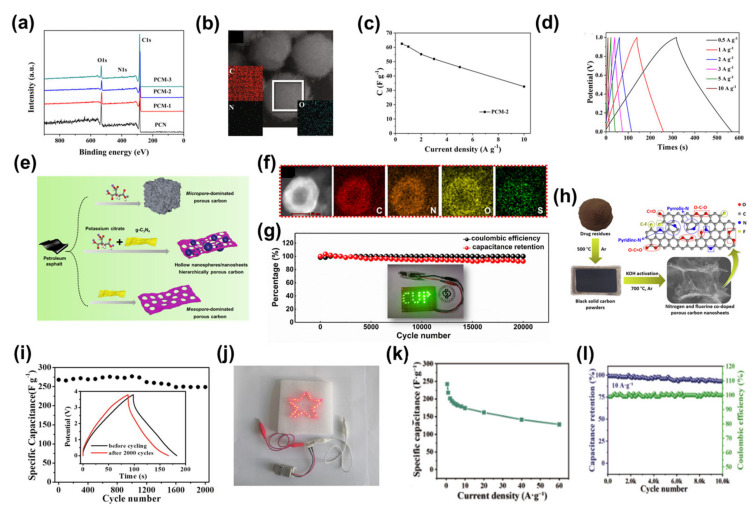
(**a**) XPS spectra of the nitrogen-doped hierarchically porous carbon microspheres. (**b**) EDS elemental mapping of the nitrogen-doped hierarchically porous carbon microspheres. (**c**) Specific capacitance of the supercapacitors based on the nitrogen-doped hierarchically porous carbon microsphere electrodes at different current densities. (**d**) GCD curves at different current densities. Reproduced with permission from Ref. [167]. Copyright 2020 The Authors. Carbon Energy published by Wenzhou University and John Wiley & Sons Australia, Ltd. (**e**) Schematic diagram of the synthesis of heteroatom-doped hierarchically porous carbon with multi-scale structure. (**f**) EDS elemental mapping of C, N, O and S in heteroatom-doped hierarchically porous carbon with multi-scale structure. (**g**) Long cycle performance of the supercapacitors based on the heteroatom-doped hierarchically porous carbon electrodes with multi-scale structure. Reproduced with permission from Ref. [170]. Copyright 2021 Elsevier Ltd. (**h**) Schematic diagram of the synthesis of 3D nitrogen-fluorine co-doped porous carbon nanosheets. (**i**) Cycle performance of the supercapacitors based on the 3D nitrogen-fluorine co-doped porous carbon nanosheet electrodes. (**j**) Supercapacitor based on the 3D nitrogen-fluorine co-doped porous carbon nanosheet electrode for powering LED lamps. Reproduced with permission from Ref. [171]. Copyright 2022 Elsevier Inc. (**k**) Variation of specific capacitance of the supercapacitor based on the nitrogen-oxygen co-doped 3D hierarchically porous carbon electrode at different current densities. (**l**) Long-cycle performance of the supercapacitors based on the nitrogen-oxygen co-doped 3D hierarchically porous carbon electrodes. Reproduced with permission from Ref. [172]. Copyright Tsinghua University Press 2022.

Shao et al. constructed nitrogen-doped hierarchically porous carbon microspheres by hydrothermal carbonisation and thermal annealing [167]. The XPS spectra showed that C (285 eV) and O (533 eV) were the major components and N (400 eV) was the minor component in all the hierarchically porous carbon microsphere samples (Figure 14a). In addition, from the EDS elemental mapping, the elements N and O were uniformly dispersed in the carbon matrix (Figure 14b). The supercapacitors based on the nitrogen-doped hierarchically porous carbon microsphere electrodes showed high specific capacitances of 62.5, 60.5, 55.2, 51.9, 46.3 and 32.6 F g^−1^ at 0.5, 1, 2, 3, 5 and 10 A g^−1^. Moreover, the shape of the charge-discharge curve of the device at different current densities is similar, which also proves that the device has good cycle stability (Figure 14c,d). In addition, the device had an energy density of up to 8.5 Wh kg^−1^ at a power density of 122.1 W kg^−1^. At power densities up to 1875.0 W kg^−1^ the energy density was still 3.4 Wh kg^−1^, comparable to that previously reported for carbon-based symmetrical supercapacitors in alkaline media [168,169]. In another example Yang et al. investigated the preparation of heteroatom-doped hierarchically porous carbon with a nanosheet/hollow-nanosphere multiscale structure using petroleum pitch as the carbon feedstock by a simple two-template strategy (Figure 14e) [170]. The EDS elemental mapping images further revealed the presence of C, N, O and S elements (Figure 14f). The abundance of heteroatoms provided sufficient active sites to optimise the capacitive properties of porous carbon. The symmetrical supercapacitor assembled from this electrode had a large energy density of 12.95 Wh kg^−1^ at a power density of 250 W kg^−1^. Impressively, the energy density was further increased to 25.5 Wh kg^−1^ at a power density of 450 W kg^−1^ using the Na_2_SO_4_ electrolyte. The devices were charged and discharged 20,000 times at a current density of 10A g^−1^ with a capacity retention rate of over 90% (Figure 14g). This work may provide new insights into the design of advanced carbon-based materials for use in supercapacitors. In addition, Pan et al. prepared 3D nitrogen and fluorine co-doped porous carbon nanosheets by high temperature pyrolysis and KOH activation using drug residues (Figure 14h), which had a high SSA of 2912 m^2^ g^−1^, a well-defined pore structure, an appropriate pore size distribution and an abundance of surface heteroatoms [171]. When examined as electrodes for supercapacitors with ionic liquids as the electrolyte, the device exhibited good cycling stability during 2000 cycles of charge and discharge at a current density of 3 A g^−1^. The specific capacitance was reduced to 249 F g^−1^ and the capacitance retention was 93% (Figure 14i). A supercapacitor based on two 3D nitrogen-fluorine co-doped porous carbon nanosheet electrodes in series can light up 40 parallel pentagram-like red light-emitting diodes (Figure 14j). All of these performances demonstrate the device’s excellent energy storage/output capability to meet the growing demand for sustainable energy storage devices. There is also a simple in situ self-templating strategy developed by Xiong et al. to prepare 3D hierarchically porous carbon with good micro- and mesoporous structures derived from a novel oxygen-bridged porous organic polymer which was prepared by the condensation of melamine and hydroquinone in an ethanolic solution of NaOH [172]. The large bounding hole structure and the abundant doping of N and O heteroatoms effectively contributed to the accessibility and electronic conductivity of the electrolyte and provided an abundance of active sites for energy storage. The supercapacitor based on the oxygen-bridged porous organic polymer electrode still had a specific capacitance of 162 F g^−1^ at a high current density of 20 A g^−1^, demonstrating excellent fast charge and discharge performance (Figure 14k). The long cycle performance of the device was also very good, with a capacitance retention rate of over 94% after 10,000 charge/discharge cycles at a current density of 10 A g^−1^ (Figure 14l). This work is instructive in fine-tuning porous structures to optimise the application of carbon materials for high-performance supercapacitors.

**Figure 15:**
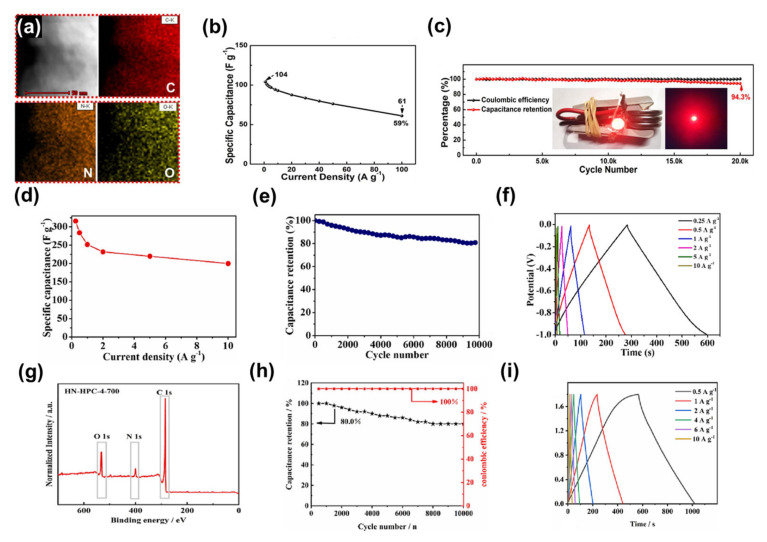
(**a**) EDS images for C, N, and O elements of the hierarchically porous carbon nanosheets. (**b**) Specific capacitance variation of the supercapacitors based on the nitrogen, oxygen co-doped layered porous carbon nanosheet electrodes at different current densities. (**c**) Long-cycle performance of the supercapacitors based on the nitrogen, oxygen co-doped layered porous carbon nanosheet electrodes and their photos for powering red LED lamps. Reproduced with permission from Ref. [173]. Copyright 2021 Published by Elsevier B.V. (**d**) Specific capacitance variation of the supercapacitors based on bamboo-derived layered porous carbon electrodes at different current densities. (**e**) Long-term cycle performance of the supercapacitors based on bamboo-derived layered porous carbon electrodes at a current density of 1 A g^−1^. (**f**) GCD curves at different current densities. Reproduced with permission from Ref. [174]. Copyright 2023 Elsevier Ltd. (**g**) XPS spectra of the nitrogen-doped porous carbon materials. (**h**) Long-term cycle performance of the supercapacitors based on the nitrogen-doped porous carbon electrodes at a current density of 10 A g^−1^. (**i**) GCD curves at different current densities. Reproduced with permission from Ref. [182]. Copyright 2023 Elsevier B.V.

Li et al. designed a simple one-step synthesis strategy to synthesise N, O co-doped hierarchically porous carbon nanosheets from g-C_3_N_4_ [173]. Both N and O were uniformly distributed throughout the carbon nanosheets as observed by X-ray energy dispersive spectroscopy (Figure 15a). The supercapacitor assembled from this electrode material had good specific capacitance at different current densities. When the current density was 1 A g^−1^, the specific capacitance of the device was 104 F g^−1^. Even at an ultrahigh current density of 100 A g^−1^, the device still exhibited a specific capacitance of 61 F g^−1^, corresponding to 59% of the initial capacitance (Figure 15b). In addition, the device also had a good long-term cycle capability. At a high current density of 10 A g^−1^, the capacity retention rate of the device was as high as 94.3% after 20,000 charge-discharge cycles (Figure 15c). In another example, Suhr et al. used ZnCl_2_/KCl salt mixture as a pore-directing solvent to directly prepare hierarchically porous carbons based on bamboo under air atmosphere through an environmentally friendly, one-step and easily scalable salt-templating strategy [174]. The porous carbon had an SSA of 1296 m^2^ g^−1^. The specific capacitances of the supercapacitors assembled from this material were 316, 284, 252, 232, 220 and 200 F g^−1^ at current densities of 0.25, 0.5, 1, 2, 5 and 10 A g^−1^, respectively. It had good specific capacitance at both low and high currents (Figure 15d). At a current density of 1 A g^−1^, the charge-discharge cycle of the device can reach 10,000 times, and the capacitance retention rate is 81%, and the shape of the charge-discharge curve of the device is similar at different current densities. All these can characteristics indicate that the device has good cycle stability (Figure 15e,f). In addition, the device had an energy density of 11 Wh kg^−1^ at a power density of 126 W kg^−1^, and an energy density of 7 Wh kg^−1^ even at a high power density of 5000 W kg^−1^. It is superior to the performance of commercial activated carbon and other biomass carbon-based symmetric supercapacitors reported by other scholars in the literature [175,176,177,178,179,180,181]. In addition, Jia et al. prepared a gel film from aspartic acid nitrate formed by aspartic acid through the study of chitosan, and prepared N, O co-doped porous carbon [182]. The prepared porous carbon had an SSA of 1599 m^2^ g^−1^. The author confirmed the existence of C, O, N elements in the material by XPS spectrum. The C, O, and N element contents were 82.27%, 10.70%, and 7.04%, respectively (Figure 15g). In addition, supercapacitors assembled from this material have good long-term cycling ability. At a high current density of 10 A g^−1^, the capacity retention is still about 80% after 10,000 charge-discharge cycles. And under different current densities, the shape of the charge-discharge curves of the device does not change greatly. (Figure 15h,i). Moreover, the device had a high energy density of 28.3 Wh kg^−1^ at a power density of 225 W kg^−1^, and still had an energy density of 20.2 Wh kg^−1^ at a power density of 4500 W kg^−1^, which is superior to that of recently reported chitosan-based electrode materials [170,183,184,185]. In addition to the above scholars, there are many examples in the research of heteroatom-doped hierarchically porous carbon materials used as supercapacitor electrodes. For example, Tong et al. prepared N/S co-doped graphene-reinforced carbon foams with ideal 3D-interconnected hierarchically porous structures for supercapacitor electrodes through a one-pot sol-gel method coupled with one-step high-temperature treatment. The as-developed porous carbon materials exhibited excellent electrochemical performance in a three-electrode system. The assembled solid-state symmetric supercapacitor delivered an energy density of 18.4 Wh kg^−1^ at 300 W kg^−1^ [186]. Liu et al. prepared N/O-doped 3D hierarchically porous sheets by high-temperature pyrolysis of poly(5-carboxybenzene-1,3-diamine disulfate). The devices based on this material exhibited only 9% capacity loss after 10,000 charge-discharge cycles at a high voltage of 4V [187]. Weng et al. reported the synthesis of N/O co-doped hierarchically porous carbon by combining urea and KOH via a one-pot and template-free method. The supercapacitors using this material as electrodes revealed excellent cycling stability, with a capacity of 240 F g^−1^ after 100,000 cycles at a current density of 20 A g^−1^ [188]. There are still more examples that are not included in this review article. Because of the presence of heteroatoms, the devices assembled using hierarchically porous carbons as supercapacitor electrodes in the above examples are all pseudocapacitor-dominated supercapacitors.

**Table 5:** Presentative applications of different types of heteroatom-doped porous carbons.

Type	Electrolyte	2 Electrode/3 Electrode System	Heteroatoms	Average Pore Size (nm)	Pore Volume (cm^3^ g^−1^)	SSA (m^2^ g^−1^)	Ref.
Heteroatom-doped microporous carbon	1 M H_2_SO_4_	3 electrodes	N, P	—	0.83	1484	[157]
	1 M H_2_SO_4_	3 electrodes	N, O	0.72	—	539	[158]
Heteroatom-doped mesoporous carbon	6 M KOH	3 electrodes	N	17.5−29.1	3.6	1037	[69]
	6 M KOH	3 electrodes	N, P, O	5−20	—	511.4	[162]
Heteroatom-doped macroporous carbon	6 M KOH	2 electrodes	N	2 × 10^5^	—	50.24	[165]
	0.8 M KPF_6_	2 electrodes	Se, N	—	—	711.86	[166]
Heteroatom-doped hierarchically porous carbon	6 M KOH	3 electrodes	N, O	1.98	1.20	1160–1791	[167]
	6 M KOH	3 electrodes	N, O, S	—	1.06	1574	[170]
	EMIMBF_4_	2 electrodes	N, F	2–10	1.95	2912	[171]
	6 M KOH, 1 M Na_2_SO_4_	2 electrodes and 3 electrodes	N, O	—	1.25	2239.5	[172]
	6 M KOH	3 electrodes	N, O	—	1.85	2557	[173]
	6 M KOH	3 electrodes	O	3.88	1.26	1296	[174]
	6 M KOH	3 electrodes	N	0.524	0.847	1599	[182]

## 4. Summary and Outlook

In summary, supercapacitors are an important energy storage device and are playing an increasingly important role in today’s energy storage applications. Finding new porous carbons that offer low cost, suitable SSA and good performance is essential for the further market penetration of supercapacitors.

Of the several methods we have described for the synthesis of porous carbons, carbonisation-physical activation is the one that has been commercialised, but the SSA of the prepared porous carbons is small. The biggest problem facing carbon-chemical activation is that some of its chemical activators are highly corrosive and even toxic, and that highly corrosive by-products and contaminants may be released during the activation process.

For the template methods, the hard-templating method is a common method for preparing porous carbons in the template methods, but its expensive inorganic template and the fact that the template cannot be easily removed are what hinders the development of this method. While both the soft-templating method and the sacrificial-template method can be used to remove the template in a simple way, the problem to be solved is how to enhance the SSA of the porous carbons obtained by these two methods. The self-templating method discards the traditional idea of preparing porous carbons on a template and uses materials such as MOFs, which are quite popular today, and low-cost citrates to generate porous carbons directly, and the resulting porous carbon materials also achieve quite high SSAs. Today there are also many new ideas for the preparation of porous carbons combining the template methods with activation, which are also expected to receive more attention and development in the future.

Whereas other emerging methods of porous carbons prepared by direct pyrolysis of block copolymers have yielded high SSA, these processes are admittedly complex and the precursors are expensive. There is therefore a need for cheaper polymer precursors, and simplified and scalable processes to make these processes a reality. The mechanisms inherent in these direct pyrolysis methods need to be further investigated in order to design better block copolymers and use them for the preparation of porous carbons. Given the abundance of carbohydrates in nature, carbohydrate self-activation will play an increasingly important role in the generation of porous carbons. However, research into this method is only in its infancy and more research is needed into the mechanisms of self-activation and how to make full use of decomposition gases (CO_2_) for the production of porous carbons. Laser scribing, on the other hand, is opening up a new avenue for graphene-based electrode materials. Not only does the porous carbon material have a high SSA, but it also has the high electrical conductivity that comes with a highly graphitised structure. This is something that is difficult to achieve with other methods.

In this review, we also list the applications of porous carbons as supercapacitor electrodes in recent years. Some evaluation of heteroatom-doped porous carbons has also been carried out. It can be seen that the microporosity in porous carbons exposes more active sites, the mesoporosity/macroporosity helps the rapid diffusion of electrolyte ions, and the hierarchical porosity, due to its 3D-interconnected structure, the combination of porosity with different pore sizes (i.e., microporous/mesoporous/macroporous) and the suitable porous distribution, is expected to favour the mass transport, buffer the volume change of the electrodes at high charging/discharging rates, and exhibit a high specific energy and good rate capability and cycling performance [132]. However, it is an undeniable fact that most hierarchically porous carbons are unable to maintain their excellent capacitance at rapid charge and discharge rates. This is particularly detrimental to supercapacitors, which are a type of charge storage device that is expected to have a high power density. How to increase the role of mesopores and macropores in facilitating ion diffusion deserves further study. In some cases, the expected characteristics may not always have a positive impact on performance. For example, heteroatom doping is commonly used to modify carbon materials, which can advantageously introduce active sites, but excessive doping can also damage the porous structure, and an excess of some atoms (e.g., oxygen) may impair the electrical conductivity of porous carbons. Overcoming the existing problems with porous carbons requires long-term efforts in both theoretical and experimental research.

In general, porous carbons have become the most important electrode material for supercapacitors. However, how to reduce the cost of preparing porous carbons, how to reduce the environmental problems caused by the process of preparing porous carbons and how to obtain better porous carbon performance will determine the future of porous carbons in supercapacitor applications.

## Data Availability

Data will be available upon request from the corresponding authors.
